# Diagnostic Performance of ^18^F-FDG PET/CT in Infectious and Inflammatory Diseases according to Published Meta-Analyses

**DOI:** 10.1155/2019/3018349

**Published:** 2019-07-25

**Authors:** Giorgio Treglia

**Affiliations:** ^1^Clinic of Nuclear Medicine and PET/CT Center, Imaging Institute of Southern Switzerland, Ente Ospedaliero Cantonale, Bellinzona and Lugano, Switzerland; ^2^Health Technology Assessment Unit, Ente Ospedaliero Cantonale, Bellinzona, Switzerland; ^3^Department of Nuclear Medicine and Molecular Imaging, Lausanne University Hospital, University of Lausanne, Lausanne, Switzerland

## Abstract

**Purpose:**

To date, several meta-analyses have reported data about the diagnostic performance of ^18^F-FDG PET/CT in infectious and inflammatory diseases. This article aims to summarize the published evidence-based data about the diagnostic performance of ^18^F-FDG PET/CT in this setting.

**Methods:**

A comprehensive computer literature search of meta-analyses published in PubMed/MEDLINE and Cochrane library database from January 2009 through December 2018 and regarding the diagnostic performance of ^18^F-FDG PET/CT in infectious and inflammatory diseases was carried out. This combination of key words was used: (i) “PET” OR “positron emission tomography” OR “FDG” OR “fluorodeoxyglucose” AND (ii) meta-analysis. Only records on inflammatory or infectious diseases were selected.

**Results:**

The diagnostic performance of ^18^F-FDG PET/CT in detecting inflammatory and infectious diseases has been summarized taking into account 36 meta-analyses published in the literature. Evidence-based data demonstrated good diagnostic performance of ^18^F-FDG PET/CT for several inflammatory and infectious diseases, in particular cardiovascular infectious and inflammatory diseases and some musculoskeletal infections.

**Conclusions:**

Evidence-based data about the diagnostic performance of ^18^F-FDG PET/CT in infectious and inflammatory diseases are increasing, with good diagnostic performance of this imaging method for some indications. More prospective multicenter studies and cost-effective analyses are warranted.

## 1. Introduction

Nuclear medicine techniques are noninvasive tools that can early detect pathophysiological changes in affected tissues in patients with inflammatory or infectious diseases. These changes usually occur before clinical onset of symptoms and before the development of anatomical changes detected by radiological techniques [1, 2]. Currently, hybrid imaging techniques as positron emission tomography/computed tomography (PET/CT) may provide functional and morphological information for early diagnosis of infectious and inflammatory diseases [1, 2].

Fluorine-18 fluorodeoxyglucose (^18^F-FDG) is a radiolabelled glucose analogue taken up by cells via cell membrane glucose transporters and subsequently phosphorylated with hexokinase inside most cells [3]. The ability of ^18^F-FDG PET/CT to identify sites of inflammation and infection is mainly related to the glycolytic activity of the cells involved in the inflammatory response [3].

**Figure 1:**
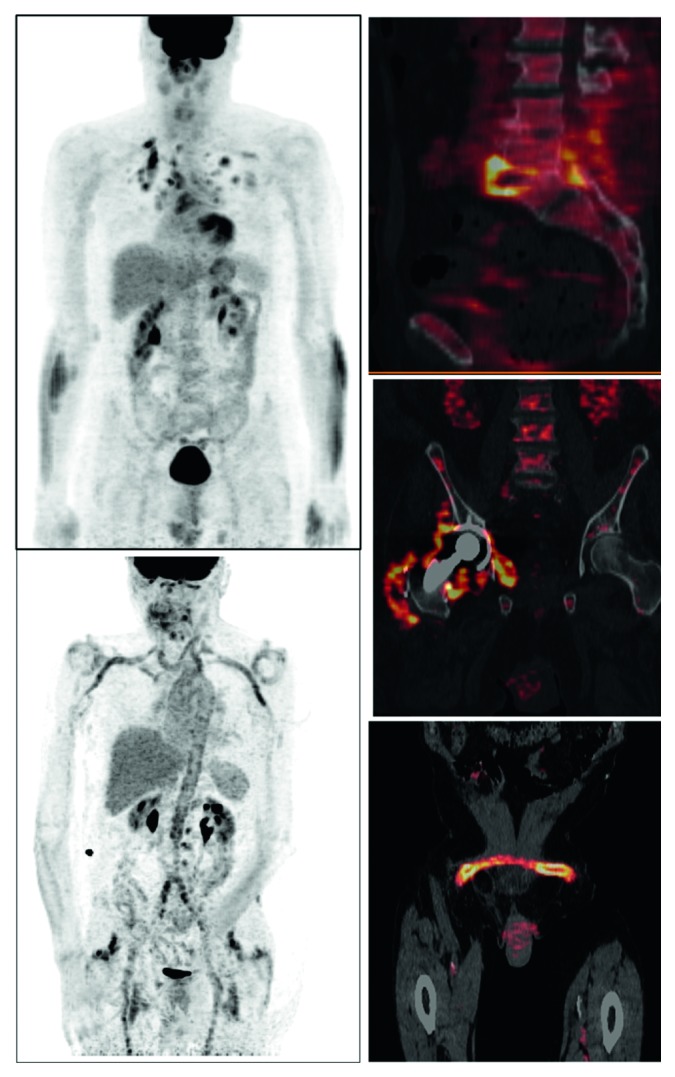
Examples of infectious and inflammatory diseases detected by ^18^F-FDG PET/CT: systemic sarcoidosis with pulmonary and mediastinal involvement (upper left), large vessel vasculitis associated with inflammatory rheumatic disease (lower left), spondylodiscitis (upper right), periprosthetic joint infection (middle right), and vascular graft infection (lower right).

^18^F-FDG PET/CT has been proposed for imaging of infectious or inflammatory diseases (Figure 1) because it has been demonstrated that cells involved in infection and inflammation, especially neutrophils and the monocyte/macrophage family, are able to express high levels of glucose transporters and hexokinase activity [3–5].

Enough evidence in the literature already exists about the usefulness of ^18^F-FDG PET/CT in the diagnosis and management of several infectious and inflammatory diseases [5]. The aim of this article is to summarize the findings of meta-analyses published in the last ten years about the diagnostic performance of ^18^F-FDG PET/CT in this setting.

## 2. Methods

A comprehensive computer literature search of PubMed/MEDLINE and Cochrane library databases was conducted to find recent published meta-analyses on the diagnostic performance of ^18^F-FDG PET/CT for the diagnosis of infectious and inflammatory diseases.

A search algorithm based on the combination of the following terms was used: (i) “PET” OR “positron emission tomography” OR “FDG” OR “fluorodeoxyglucose” AND (ii) meta-analysis. The literature search was updated until December 31^st^, 2018. No language restriction was used. Recent meta-analyses published in the last ten years and investigating the diagnostic performance of ^18^F-FDG PET/CT in infectious or inflammatory diseases were eligible for inclusion. Titles and abstracts of the retrieved articles were reviewed, applying the inclusion criteria mentioned above.

For each selected meta-analysis, information was collected about basic study characteristics (disease evaluated, authors, year of publication, number of original articles included, and number of patients included) and pooled diagnostic performance measures including 95% confidence interval values (95% CI).

Main findings of the selected meta-analyses were briefly described.

## 3. Results

From the comprehensive computer literature search from PubMed/MEDLINE and Cochrane databases, 36 meta-analyses were selected and retrieved in full-text version [6–41]. The characteristics of the selected articles are presented in Table 1 and summarized here below.

### 3.1. Fever of Unknown Origin (FUO)

Fever of unknown origin (FUO) is a very precise entity, as described in the literature. FUO is commonly defined as temperature ≥38.3°C on at least two occasions, duration of illness ≥3 weeks or multiple febrile episodes in ≥3 weeks, not immunocompromised patient, and uncertain diagnosis despite thorough history-taking, physical examination, and obligatory investigations [42]. The diagnosis in patients with FUO is a challenging medical problem; the causes of FUO may be infectious diseases, noninfectious inflammatory diseases, or tumours, and ^18^F-FDG PET/CT detecting foci of increased glucose metabolism may be used for revealing the source of fever [42]. Several meta-analyses have estimated the diagnostic performance of ^18^F-FDG PET/CT in the assessment of FUO unidentified by conventional workup [6–12].

Dong et al. firstly reported that the pooled sensitivity and specificity of ^18^F-FDG PET/CT for the detection of FUO were 98.2% (95% CI: 93.6–99.8) and 85.9% (95% CI: 75–93.4), respectively. Therefore, this method should be considered among the first diagnostic tools for patients with FUO in whom conventional diagnostics have been unsuccessful [6].

Hao et al. confirmed the high sensitivity of ^18^F-FDG PET/CT for the diagnosis of patients with FUO (pooled value: 85%; 95% CI: 81–88), but the possibility of false-positive results should be kept in mind [7].

Another meta-analysis demonstrated that abnormal ^18^F-FDG PET/CT findings are associated with a substantially increased final diagnostic rate in FUO (pooled odds ratio: 8.94; 95% CI: 4.18–19.12, *p* < 0.00001). Consequently, ^18^F-FDG PET/CT could be considered for inclusion in the first-line diagnostic workup of FUO. Further randomized prospective studies with standardized ^18^F-FDG PET/CT are warranted to confirm this first-line position [8].

Tateuchi et al. reported that ^18^F-FDG PET/CT can be useful in identifying the source of fever in patients with classic FUO (immunocompetent patients). The summary sensitivity, specificity, and diagnostic yield of this method were 86% (95% CI: 81–90), 52% (95% CI: 36–67), and 58% (95% CI: 51–64), respectively. The contribution of ^18^F-FDG PET/CT may be limited in clinical settings in which infectious and neoplastic causes are less common. Indirect comparisons of test performance suggested that ^18^F-FDG PET/CT outperformed standalone ^18^F-FDG PET, gallium-67 scintigraphy, and radiolabelled leukocyte scintigraphy in detecting causes of FUO. Studies using standardized diagnostic algorithms are needed to determine the optimal timing for testing and to assess the impact of tests on management decisions and patient-relevant outcomes [9].

Recently, Bharucha et al. reported an overall diagnostic contribution of 56% (95% CI: 50–61) of ^18^F-FDG PET/CT in all patients with FUO. In a subgroup analysis taking into account previous investigations, the diagnostic yield/added contribution of ^18^F-FDG PET/CT over CT was 32% (95% CI: 22–44). The pooled proportion of abnormal ^18^F-FDG PET/CT in patients with FUO was 69% (95% CI: 63–75); the higher proportion of abnormal scans was accounted for by a proportion of false-positive abnormal scans with no contribution to the final diagnosis, with an overall result of 9% (95% CI: 5–14). The authors concluded that there is insufficient evidence to support the value of ^18^F-FDG PET/CT in investigative algorithms of FUO [10].

Conversely, in an updated meta-analysis on patients with FUO or inflammation of unknown origin (IUO), ^18^F-FDG PET/CT was demonstrated to be very helpful for recognizing and excluding diseases, directing further diagnostic decisions and avoiding unnecessary invasive examinations. The pooled sensitivity and specificity were 84% (95% CI: 79–89) and 63% (95% CI: 49–75), respectively. Based on these findings, the authors recommended ^18^F-FDG PET/CT among the first-line diagnostic tools for patients with FUO and IUO [11].

Lastly, it has been recently demonstrated that patients with negative ^18^F-FDG PET/CT results were significantly more likely to present with spontaneous fever regression than those with positive ^18^F-FDG PET/CT results (summary relative risk = 5.6 : 95% CI: 3.4–9.2; *p* < 0.001) [12].

Overall, there is no agreement among the selected meta-analyses about the added value of ^18^F-FDG PET/CT in patients with FUO. The main drawback of the meta-analyses evaluating the diagnostic performance of ^18^F-FDG PET/CT for this specific indication is that they include articles without real FUO patients or with highly variable definitions of FUO; therefore, related meta-analyses could be not accurate in this regard [43].

Furthermore, the diagnostic yield of ^18^F-FDG PET/CT in patients with FUO should take into account not only the positive cases but also the true negative cases as patients with a negative ^18^F-FDG PET/CT are likely to have a favourable course [12]. Considering that 30–50% of patients with FUO will not have a final diagnosis and do well after prolonged follow-up without further treatment, the diagnostic yield of ^18^F-FDG PET/CT might be even higher compared to that reported in the selected meta-analyses [43].

### 3.2. Large Vessel Vasculitis (LVV)

Large vessel vasculitis (LVV) is defined as an inflammatory disease mainly affecting the large arteries, with two major variants, Takayasu arteritis (TA) and giant cell arteritis (GCA). GCA often coexists with polymyalgia rheumatica (PMR) in the same patient, since both belong to the same disease spectrum [44]. ^18^F-FDG PET/CT may demonstrate increased radiopharmaceutical uptake in the vascular wall of large vessels in patients with LVV; therefore, this method may be used for diagnosis, monitoring of disease activity, and evaluating disease progression in LVV [44–47], and several meta-analyses have assessed the role of this imaging method in this setting [13–19].

First meta-analyses including both ^18^F-FDG PET and PET/CT studies reported a valuable diagnostic performance of these methods in patients with GCA with a pooled sensitivity and specificity of 80% (95% CI: 63–91) and 89% (95% CI: 78–94), respectively [13], and a moderate value of these methods in assessing TA activity, with a pooled sensitivity and specificity of 70.1% (95% CI: 58.6–80) and 77.2% (95% CI: 64.2–87.3), respectively [14].

In a meta-analysis of Soussan et al. including both ^18^F-FDG PET and PET/CT studies, these imaging methods showed good performances in the diagnosis of LVV, with higher accuracy in GCA patients than in TA patients. A vascular uptake equal to or higher than the liver uptake appeared to be a good criterion for the diagnosis of vascular inflammation. ^18^F-FDG PET or PET/CT showed high sensitivity and specificity for the diagnosis of LVV in GCA patients in comparison to controls, with pooled values of 90% (95% CI: 79–93) and 98% (95% CI: 94–99), respectively. ^18^F-FDG PET or PET/CT had a pooled sensitivity of 87% (95% CI: 78–93) and specificity of 73% (95% CI: 63–81) for the assessment of disease activity in TA, with up to 84% of specificity in studies using National Institutes of Health criteria as the disease activity assessment scale [15].

Another meta-analysis by Lee et al. confirmed that ^18^F-FDG PET/CT has good diagnostic accuracy for LVV with a pooled sensitivity and specificity of 83.9 % (95% CI: 71.7–92.4) and 87.2% (95% CI: 72.6–95.7), respectively [16].

In a recent meta-analysis, the pooled sensitivity and specificity of ^18^F-FDG PET or PET/CT for detecting active disease in TA compared to clinical assessment were 81% (95% CI: 69–89) and 74% (95% CI: 55–86), respectively. Active disease by ^18^F-FDG PET or PET/CT was also associated with elevations of acute phase reactants, as C-reactive protein (CRP) and erythrocyte sedimentation rate (ESR) [17]. Conversely, in another meta-analysis by Gomez et al. about the association between the CRP value and ^18^F-FDG PET or PET/CT vascular positivity in TA, CRP concentration only moderately reflected the ^18^F-FDG PET vascular positivity in TA, suggesting dissociated information [18]. More prospective studies are needed to assess the value of ^18^F-FDG PET/CT as an independent biomarker for subtle vascular wall inflammation detection in patients with TA [18].

Lastly, an updated meta-analysis confirmed that ^18^F-FDG PET or PET/CT has a good performance for the detection of active disease in patients with LVV with a pooled sensitivity and specificity of 88% (95% CI: 79–93) and 81% (95% CI: 64–91), respectively. Therefore, ^18^F-FDG PET/CT could be suggested as a surrogate biomarker for assessment of disease activity of LVV during or after immunosuppressive therapy, but further studies are warranted to determine if PET-based treatment of LVV can improve outcomes [19].

Several factors may significantly influence the arterial wall ^18^F-FDG uptake and must be taken into consideration for interpretation of ^18^F-FDG PET/CT in LVV [44]. Many PET interpretation criteria have been proposed; nevertheless, evidence supports the use of a visual ^18^F-FDG PET grading scale with vascular ^18^F-FDG uptake ≥ liver uptake as LVV positivity criterion [44]. Atherosclerotic vascular uptake may be a source of false positivity for LVV evaluation, despite a classical patchy uptake pattern. Taking these considerations into account, vascular inflammation in LVV on ^18^F-FDG PET classically appears as a smooth linear pattern [44].

Patients with suspected LVV often immediately receive high-dose glucocorticoids before ^18^F-FDG PET/CT, and this may reduce the intensity of arterial ^18^F-FDG uptake. The accuracy of ^18^F-FDG PET/CT can therefore vary in relation to the delay between the initiation of immunosuppressive therapy and ^18^F-FDG PET/CT [44].

For the precise evaluation of diagnostic accuracy of ^18^F-FDG PET/CT in patients with LVV, it should be taken into account that in some patients, ^18^F-FDG PET/CT may be the only modality allowing for the diagnosis of LVV, and therefore it cannot be compared to a gold standard [44].

Overall, based on the available evidence, ^18^F-FDG PET/CT has demonstrated high diagnostic performance for the detection of LVV. Further studies are needed to select the most clinically relevant and reproducible criteria for defining the presence of LVV with ^18^F-FDG PET/CT, as well as to test the clinical impact of ^18^F-FDG PET/CT on the management of patients with suspected LVV [44].

### 3.3. Infective Endocarditis (IE) and Cardiovascular Implantable Electronic Device (CIED) Infections

Infective endocarditis (IE) is a serious and potentially life-threatening condition. The current diagnosis of IE is based on the modified Duke criteria, which has approximately 80% sensitivity for the diagnosis of native valve endocarditis (NVE), with lower sensitivity for the diagnosis of prosthetic valve endocarditis (PVE) and culture-negative endocarditis [48, 49]. Noninvasive imaging modalities may improve diagnosis of infective endocarditis (IE) [48, 49]. In particular, ^18^F-FDG PET/CT is currently included as diagnostic tool in the diagnostic flow chart for IE [48–51], and some meta-analyses have evaluated the diagnostic performance of this method in patients with IE or CIED infections [20–24].

A first meta-analysis published in 2016 demonstrated that the overall diagnostic performance of ^18^F-FDG PET/CT for the diagnosis of IE was not high due to the low sensitivity: pooled sensitivity and specificity were 61% (95% CI: 52–88) and 88% (95% CI: 80–93), respectively. However the diagnostic performance of ^18^F-FDG PET/CT increased in the subgroup of patients with PVE [20].

Mahmood et al. demonstrated that ^18^F-FDG PET/CT may be a useful adjunctive diagnostic tool in the evaluation of diagnostically challenging cases of IE, particularly in PVE. The pooled sensitivity and specificity of ^18^F-FDG PET/CT for diagnosis of IE were 76.8% (95% CI: 71.8–81.4) and 77.9% (95% CI: 71.9–83.2), respectively. Diagnostic accuracy was improved for PVE with pooled sensitivity of 80.5% (95% CI: 74.1–86) and pooled specificity of 73.1% (95% CI: 63.8–81.2). More recent studies published from 2015 to 2017 reported a higher pooled sensitivity of 81.3% (95% CI: 74.3–87) and specificity of 79% (95% CI: 71.2–85.5). The majority of the recent studies were prospective and used a specific protocol (i.e., a low-carbohydrate fat-allowed diet for at least 24 hours prior to imaging, a prolonged fasting prior to imaging, and/or an intravenous heparin bolus prior to ^18^F-FDG administration). ^18^F-FDG PET/CT also has the potential to detect clinically relevant extracardiac foci of infection, malignancy, and other sources of inflammation leading to more appropriate treatment regimens and surgical intervention. Additional extracardiac foci of infection were found on 17% of patients in this meta-analysis [21].

In another meta-analysis, Juneau et al. demonstrated that ^18^F-FDG PET/CT has a good diagnostic accuracy for the diagnosis of IE if adequate patient preparation for suppression of physiological myocardial ^18^F-FDG uptake was performed, including prolonged fasting at least 12 hours and/or heparin injection before ^18^F-FDG administration and/or high-fat carbohydrate-restricted protein-permitted diet. Pooled sensitivity of ^18^F-FDG PET/CT performed with adequate cardiac preparation for the diagnosis of IE was 81% (95% CI: 73–86) and pooled specificity was 85% (95% CI: 78–91). In the subgroup of patients with PVE, the pooled sensitivity was 85% (95% CI: 77–91) but specificity was 81% (95% CI: 72–88). Therefore, ^18^F-FDG PET/CT may be useful in the investigation of IE and should be considered in cases where the diagnosis is uncertain [22].

^18^F-FDG PET/CT may be helpful in the diagnosis of CIED infections, particularly in patients with the absence of localizing signs or definitive echocardiographic findings. In a recent meta-analysis, Mahmood et al. reported a pooled sensitivity and specificity of ^18^F-FDG PET/CT in the diagnosis of CIED infections of 85% (95% CI: 80–89) and 90% (95% CI: 84–94), respectively. ^18^F-FDG PET/CT demonstrated a higher sensitivity of 96% (95% CI: 86–99) and specificity of 97% (95% CI: 86–99) for diagnosis of pocket infections. Diagnostic accuracy for lead infections or CIED-IE was lower with pooled sensitivity of 76% (95% CI: 65–85) and specificity of 83% (95% CI: 72–90). In the subgroup of studies that described use of any myocardial suppression protocol, the pooled sensitivity was 92% (95% CI: 85–96) and the pooled specificity was 81% (95% CI: 71–89) [23].

Another recent meta-analysis confirmed the high diagnostic performance of ^18^F-FDG PET/CT for the diagnosis of CIED infections with a pooled sensitivity of 87% (95% CI: 82–91) and a pooled specificity of 94% (95% CI: 88–98). Pooled sensitivity and specificity for diagnosis of pocket/generator related CIED infections were 93% (95% CI: 84–98) and 98% (95% CI: 88–100), respectively. Pooled sensitivity and specificity for diagnosis of lead or IE-related CIED infection were 65% (95% CI: 53–76) and 88% (95% CI: 77–94), respectively [24].

Overall, ^18^F-FDG PET/CT demonstrated a good diagnostic performance in patients with IE and CIED infections with higher diagnostic accuracy if adequate patient preparation for suppression of physiological myocardial ^18^F-FDG uptake was performed.

### 3.4. Vascular Graft Infection (VGI)

Vascular graft infection (VGI), a serious complication in vascular surgery, has a high morbidity and mortality rate. The diagnosis is complicated by nonspecific symptoms and challenged by the variable accuracy of different imaging techniques [25, 52]. A recent meta-analysis demonstrated a good diagnostic performance of ^18^F-FDG PET/CT in patients with VGI with a pooled sensitivity and specificity of 95% (95% CI: 87–99) and 80% (95% CI: 69–89), respectively [25].

Another recent meta-analysis investigating the diagnostic accuracy of ^18^F-FDG PET/CT in VGI reported a pooled sensitivity and specificity for focal ^18^F-FDG uptake of 97% (95% CI: 89–99) and 89% (95% CI: 70–96), respectively [26].

One of the factors influencing the ^18^F-FDG uptake in patients with suspicious VGI is the time at which ^18^F-FDG PET/CT is performed after surgery. In fact, ^18^F-FDG uptake reaches its peak in the first few weeks after surgery and tends towards normal values around 4 weeks postoperatively, even if long-lasting physiologic activity has also been described. If ^18^F-FDG PET/CT is performed in cases of recently implanted grafts, increased ^18^F-FDG uptake can occur in uninfected grafts leading to false-positive ^18^F-FDG PET/CT findings for VGI [26].

False-negative ^18^F-FDG PET/CT findings in VGI may occur mainly because of the use of antibiotics prior to imaging, thus lowering the metabolic activity expected in infections and lowering the ^18^F-FDG uptake [26].

Variable interpretation criteria have been used to assess VGI by ^18^F-FDG PET/CT. The sensitivity and specificity of ^18^F-FDG PET/CT may therefore vary based on the criteria used [26]. A consensus about the parameters used for interpretation of the results would lead to better diagnostic accuracy, and this could be increased by performing the scan prior to antimicrobial treatment. Results from larger prospective studies are warranted [26].

### 3.5. Sarcoidosis

Sarcoidosis is a multisystem chronic inflammatory disease of unknown etiology characterized by widespread growth of noncaseating granulomas. The diagnosis of sarcoidosis is based on clinical and imaging presentation, histological confirmation, and the absence of alternative diseases. Imaging techniques may play a role in the diagnostic workup of patients with sarcoidosis to assess disease extent and activity and treatment response evaluation [53]. The role of ^18^F-FDG PET/CT in patients with sarcoidosis is well established [54, 55]. Based on evidence-based data, the recommendations for use of ^18^F-FDG PET/CT in patients with sarcoidosis could be the following: evaluation of inflammatory active disease in patients with persistent symptoms and negative serologic markers; assessment of inflammation in radiologic stage IV sarcoidosis with lung fibrosis; evaluation of inflammatory active extrathoracic sites of sarcoidosis or assessment of cardiac sarcoidosis (especially in patients with implanted pacemakers); identification of active sites for diagnostic biopsy not revealed by other methods; and evaluation of treatment response in refractory sarcoidosis [54].

The role of ^18^F-FDG PET/CT in cardiac sarcoidosis is currently under active investigation [56], and some meta-analyses have addressed the diagnostic performance of ^18^F-FDG PET/CT in this setting [27–29].

In the meta-analysis of Youssef et al., the pooled sensitivity and specificity of ^18^F-FDG PET or PET/CT for diagnosis of cardiac sarcoidosis were 89% (95% CI: 79–96) and 78% (95% CI: 68–86), respectively [27].

Tang et al. demonstrated that the diagnostic accuracy of ^18^F-FDG PET/CT for cardiac sarcoidosis depends on adequate suppression of physiological cardiac glucose uptake. Overall, ^18^F-FDG PET/CT had a pooled sensitivity of 75% (95% CI: 69–80) and a pooled specificity of 81% (95% CI: 76–85) for the diagnosis of cardiac sarcoidosis. This modest diagnostic accuracy was attributed to the inclusion of studies in which a short fasting duration before scanning likely influenced its sensitivity. Excluding studies without adequate myocardial suppression resulted in a pooled sensitivity of 81% (95% CI: 76–86) and a pooled specificity of 82% (95% CI: 77–86). Fasting for at least 12 hours before scanning or a high-fat low-carbohydrate diet given at 3 to 6 hours before imaging or heparin infusion before imaging has shown to improve the diagnostic accuracy of ^18^F-FDG PET/CT in cardiac sarcoidosis [28].

Lastly, an updated meta-analysis on the diagnostic performance of ^18^F-FDG PET or PET/CT in cardiac sarcoidosis demonstrated a pooled sensitivity and specificity of 84% (95% CI: 71–91) and 83% (95% CI: 74–89), respectively. The presence of combined myocardial perfusion imaging improved the diagnostic accuracy of ^18^F-FDG PET/CT for diagnosis of cardiac sarcoidosis. Nevertheless, further large multicenter studies in this setting are needed [29].

### 3.6. Musculoskeletal Infections

Musculoskeletal infections are a serious problem in healthcare. Establishing the correct diagnosis is often difficult and may have a huge impact on daily life. Treatment of a musculoskeletal infection often requires a long time and/or costly procedures which can be avoided if musculoskeletal infection is excluded [57]. On the other hand, timely identification and precise localization of musculoskeletal infections by imaging techniques are critical for early initiation of treatment and can have a significant impact on patient outcome. In this setting, nuclear medicine and radiological imaging are complementary techniques [57]. In particular, several meta-analyses have investigated the diagnostic performance of ^18^F-FDG PET/CT in patients with suspicious musculoskeletal infections [30–38].

Wang et al. calculated the diagnostic performance of ^18^F-FDG PET or PET/CT in patients with suspicious osteomyelitis reporting a high pooled sensitivity and specificity in this setting: pooled values were 92.3% (95% CI: 86.7–96.1) and 92% (95% CI: 87–95.6), respectively [30].

A first meta-analysis focused on the diagnostic performance of ^18^F-FDG PET or PET/CT in osteomyelitis related to diabetic foot reported a pooled sensitivity and specificity of 74% (95% CI: 60–85) and 91% (95% CI: 85–96), respectively [31]. An updated meta-analysis on the same topic demonstrated a pooled sensitivity of 89% (95% CI: 68–97) and a pooled specificity of 92% (95% CI: 85–96) [32].

Jin et al. calculated the diagnostic performance of ^18^F-FDG PET or PET/CT in detecting prosthetic infection after arthroplasty. They found a pooled sensitivity and specificity of 86% (95% CI: 82–90) and 86% (95% CI: 83–89), respectively. The pooled sensitivity of ^18^F-FDG PET or PET/CT in demonstrating hip and knee prosthetic infection was 88% (95% CI: 83–92) and 72% (95% CI: 58–84), respectively. The pooled specificity of ^18^F-FDG PET or PET/CT in demonstrating hip and knee prosthetic infection was 88% (95% CI: 84–91) and 80% (95% CI: 71–88), respectively [33].

A meta-analysis focused on periprosthetic hip infection confirmed the good diagnostic accuracy of ^18^F-FDG PET or PET/CT in this setting with pooled sensitivity and specificity of 86% (95% CI: 80–90) and 93% (95% CI: 90–95), respectively, using increased ^18^F-FDG uptake in the bone-prosthesis interface as the criterion for infection for the index test [34].

A meta-analysis focused on periprosthetic knee infection demonstrated a not optimal diagnostic accuracy of ^18^F-FDG PET or PET/CT in this setting with pooled sensitivity and specificity of 70% (95% CI: 56–81) and 84% (95% CI: 76–90) [35].

Some factors influencing the diagnostic performance of ^18^F-FDG PET/CT in patients with osteomyelitis should be underlined: first of all, several interpretation criteria of ^18^F-FDG PET have been used in the literature, by using visual and/or semiquantitative criteria, leading to different diagnostic accuracy values [30–35]. Furthermore, continuous physiologic ^18^F-FDG activity around the prostheses may be the cause of false-positive ^18^F-FDG PET/CT findings for periprosthetic infection [33–35].

^18^F-FDG PET or PET/CT has an excellent diagnostic performance in detecting infectious spondylodiscitis [58]. A first meta-analysis on ^18^F-FDG PET or PET/CT in patients with suspicious spondylodiscitis reported a pooled sensitivity and specificity of 97% (95% CI: 83–100) and 88% (95% CI: 74–95), respectively [36]. In this setting, the diagnostic performance of ^18^F-FDG PET or PET/CT was higher compared with magnetic resonance imaging (MRI). Considering studies comparing ^18^F-FDG PET or PET/CT and MRI, pooled sensitivity and specificity of ^18^F-FDG PET or PET/CT were 96% (95% CI: 84–99) and 90% (95% CI: 79–96), whereas the pooled sensitivity and specificity of MRI were 76% (95% CI: 65–84) and 62% (95% CI: 45–77) [37]. Another recent meta-analysis confirmed the better diagnostic accuracy of ^18^F-FDG PET or PET/CT compared to MRI for the detection of spondylodiscitis: for ^18^F-FDG PET or PET/CT, pooled sensitivity and specificity were 95% (95% CI: 87–98) and 88% (95% CI: 73–95), respectively; and for MRI, pooled sensitivity and specificity were 85% (95% CI: 65–95) and 66% (95% CI: 48–80), respectively [38].

Overall, based on the available evidence, ^18^F-FDG PET/CT has demonstrated a good diagnostic performance for the detection of musculoskeletal infections.

### 3.7. Inflammatory Rheumatic Diseases

Molecular imaging methods, including ^18^F-FDG PET/CT, have been proposed for a better assessment of inflammatory rheumatic diseases [59]. ^18^F-FDG uptake in the shoulders or hips was often reported in PMR (pooled prevalence: 76%), especially in periarticular sites (pooled prevalence: 84%). Furthermore, interspinous ^18^F-FDG uptake, demonstrating interspinous bursitis, is common in PMR (pooled prevalence: 67%). However, these findings are not very specific for PMR [39].

Patients with rheumatoid arthritis (RA) may also have interspinous ^18^F-FDG uptake (pooled prevalence: 34%) or articular ^18^F-FDG uptake in shoulders or hips (pooled prevalence: 66%) or in other articular regions (pooled prevalence: 78%). Articular ^18^F-FDG uptake is not specific for PMR or RA as it is common in other connective tissue diseases (pooled prevalence: 70%). Overall, ^18^F-FDG PET/CT is helpful in diagnostic research, but the interpretation of ^18^F-FDG uptake at each site is not characteristic of a specific inflammatory rheumatic disease [39].

### 3.8. Inflammatory Bowel Diseases

^18^F-FDG PET/CT may also be used to image areas of active inflammation, such as those occurring in patients with active inflammatory bowel disease (IBD) as Crohn's disease and ulcerative colitis [60]. In this setting, ^18^F-FDG PET or PET/CT showed a good accuracy with a pooled sensitivity and specificity of 85% (95% CI: 81–88) and 87% (95% CI 84–90), respectively [40]. These findings were confirmed by another meta-analysis including prospective studies only [41]. Nevertheless, more prospective studies evaluating the role of ^18^F-FDG PET/CT for this indication are needed. Specific challenges for the use of ^18^F-FDG PET/CT in IBD are the physiological ^18^F-FDG uptake in the bowel and the movement of the bowel that may influence a correct coregistration of ^18^F-FDG PET and CT images [40].

## 4. Discussion

Overall, this article demonstrates that there is increasing evidence about the diagnostic performance of ^18^F-FDG PET/CT in infectious and inflammatory diseases, with good diagnostic accuracy values for some indications (Table 1). Awareness of the results described in this review has the potential to affect patient care by providing supportive evidence for more effective use of ^18^F-FDG PET/CT in the diagnosis of some infectious or inflammatory diseases. ^18^F-FDG PET/CT may potentially be useful to direct therapeutic strategies improving patient outcome, but prospective outcome studies are needed in this setting. In fact, diagnostic accuracy of a test is not a measure of clinical effectiveness, and good diagnostic performance does not necessarily result in improved patient outcomes. Other factors beyond the diagnostic performance should influence the choice of an imaging modality in patients with infectious and inflammatory diseases (i.e., availability, radiation dose, safety, examination time, legal, organization and economic aspects, and cost-effectiveness).

Some limitations of the included meta-analyses should be underlined because they could limit definitive conclusions on the diagnostic performance of ^18^F-FDG PET/CT in infectious and inflammatory diseases. First of all, in some meta-analyses, a limited number of published articles (some of them with small sample size) were included reducing the statistical power of the analysis (Table 1).

In several meta-analyses, a considerable heterogeneity of diagnostic performance of ^18^F-FDG PET/CT among studies was found. Heterogeneity may represent a potential source of bias in a meta-analysis. This heterogeneity is likely to arise through baseline differences among the patients in the included studies, diversity in methodological aspects between different studies, and different study quality [61]. The diversity of the interpretation criteria used for ^18^F-FDG PET/CT may also have contributed to the heterogeneity among studies.

About the study design, only some articles included in each meta-analysis were prospective studies, influencing the overall quality of the meta-analysis and causing heterogeneity. Many of the included studies are small, single-centre, retrospective series limiting their applicability to a broader setting. In most of the included studies, the ^18^F-FDG PET/CT results were available to the clinicians caring for patients which may have influenced their decision making. Moreover, interpretation of imaging was often not blinded to the clinical scenario, which may have influenced reporting of ^18^F-FDG PET/CT results.

The lack of a reliable “gold standard” for the diagnosis of infection and inflammation could be another limitation of the described meta-analyses. The studies included in the meta-analyses used multiple and imperfect reference standards and were deemed likely to have produced biased results because of differential verification and incorporation of the ^18^F-FDG PET/CT result in the reference standard. Differential verification and incorporation bias are likely to lead to overestimation of test performance, and thus the summary estimates should be interpreted with caution [61].

Also, publication bias is a major concern in all meta-analyses as studies reporting significant findings are more likely to be published than those reporting nonsignificant results [61].

Some current indications of ^18^F-FDG PET/CT in infectious and inflammation were not evaluated by meta-analyses. In particular, septicemia and/or bacteremia of unknown origin is an important current field of use of ^18^F-FDG PET/CT [62–67]; furthermore, the diagnostic performance of ^18^F-FDG PET/CT for infections caused by specific pathogens (i.e., fungal infections, mycobacteriosis, etc.) [68, 69] needs also to be evaluated through a meta-analytic approach.

Large multicenter prospective studies and in particular more cost-effective analyses comparing ^18^F-FDG PET/CT with other imaging modalities in infectious and inflammatory diseases are warranted. To this regard, cost-effective analyses on ^18^F-FDG PET/CT in patients with FUO and bacteremia are already available, demonstrating that ^18^F-FDG PET/CT appears to be a cost-effective imaging technique in these settings by avoiding unnecessary investigations and reducing the duration of hospitalization [70–73].

By using ^18^F-FDG PET/CT, it is difficult to distinguish sterile inflammation from inflammation caused by infection. Even though radiolabelled leukocytes are routinely used in clinical practice for this purpose, new radiopharmaceuticals including imaging agents targeting bacteria are currently investigated [74, 75]. A recent evidence-based review highlighted the availability of many promising PET radiopharmaceuticals for bacterial imaging despite some bias related to animal selection and index test, but few have been translated to human subjects. Results showed a lack of standardized infection models and experimental settings [75]. PET/CT using autologous leukocytes radiolabelled with ^18^F-FDG demonstrated a good diagnostic accuracy for the diagnosis of infectious diseases, but larger studies are needed [76].

Furthermore, radiopharmaceuticals beyond ^18^F-FDG have been developed for evaluating inflammatory diseases, in particular to define new strategies for imaging immune cells as well as tissue modifications induced by the inflammatory process [77].

The role of ^18^F-FDG PET/CT for assessing treatment response in infectious or inflammatory diseases is also promising [78]. However, there are currently no recommended imaging modalities to objectively evaluate the effectiveness of treatment. Therapeutic effectiveness is currently gauged by the patient's subjective clinical response [78].

Hybrid PET/MRI systems, combining functional information with high soft tissue contrast provided by MRI, are now available for clinical use. Although the role of ^18^F-FDG PET/MRI in infectious and inflammatory diseases is promising, these areas of clinical investigation are still in the early phase, and more evidence-based data are needed in this setting [79].

## Figures and Tables

**Table 1:** Characteristics and main findings of included meta-analyses on the diagnostic performance of ^18^F-FDG PET/CT in infectious or inflammatory diseases.

Topic	Authors	Year	Articles included about ^18^F-FDG PET/CT	Patients included	Sensitivity (95% CI)	Specificity (95% CI)	LR+ (95% CI)	LR− (95% CI)	DOR (95% CI)
Fever of unknown origin	Dong et al. [6]	2011	4	174	98.2% (93.6–99.8)	85.9% (75.0–93.4)	5.8 (3.3–10)	0.05 (0.01–0.25)	7.1 (0.7–67.4)
	Hao et al. [7]	2013	15	595	85% (81–88)	NR	NR	NR	NR
	Besson et al. [8]	2016	7	401	NR	NR	NR	NR	NR
	Takeuchi et al. [9]	2016	22	1137	86% (81–90)	52% (36–67)	NR	NR	NR
	Bharucha et al. [10]	2017	18	905	NR	NR	NR	NR	NR
	Kan et al. [11]	2018	23	1927	84% (79–89)	63% (49–75)	2.3 (1.5–3.4)	0.25 (0.16–0.38)	9 (4–20)
	Takeuchi et al. [12]	2018	9	418	NR	NR	NR	NR	NR

Large vessel vasculitis	Besson et al.^*∗*^ [13]	2011	6	283	[GCA] 80% (63–91)	[GCA] 89% (78–94)	[GCA] 6.73 (3.5–12.8)	[GCA] 0.25 (0.13–0.46)	NR
	Cheng et al.^*∗*^ [14]	2013	6	142	[TA] 70.1% (58.6–80)	[TA] 77.2% (64.2–87.3)	[TA] 2.3 (1.1–4.8)	[TA] 0.34 (0.14–0.82)	[TA] 7.5 (1.6–34)
	Soussan et al.^*∗*^ [15]	2015	21	712	[GCA] 90% (79–96)	[GCA] 98% (94–99)	[GCA] 28.7 (11.5–71.6)	[GCA] 0.15 (0.07–0.29)	[GCA] 256.3 (70.8–927)
					[TA] 87% (78–93)	[TA] 73% (63–81)	[TA] 4.2 (1.5–12)	[TA] 0.2 (0.1–0.5)	[TA] 19.8 (4.5–87.6)
					[TA^+^] 84% (73–92)	[TA^+^] 84% (73–92)	[TA^+^] 4.6 (2.1–9.9)	[TA^+^] 0.2 (0.1–0.5)	[TA^+^] 23.4 (5.2–105.2)
	Lee et al. [16]	2016	3	95	83.9% (71.7–92.4)	87.2% (72.6–95.7)	5.2 (2.4–11.2)	0.2 (0.1–0.4)	27.2 (8.5–86.6)
	Barra et al.^*∗*^ [17]	2018	10	301	[TA] 81% (69–89)	[TA] 74% (55–86)	NR	NR	NR
	Gomez et al.^*∗*^ [18]	2018	9	210	NR	NR	NR	NR	NR
	Lee et al.^*∗*^ [19]	2018	9	298	88% (79–93)	81% (64–91)	4.5 (2.2–9.5)	0.15 (0.08–0.29)	30 (8–107)

Infective endocarditis	Yan et al. [20]	2016	6	246	61% (52–88)	88% (80–93)	3.24 (1.67–6.28)	0.5 (0.32–0.77)	6.98 (2.5–19.1)
	Mahmood et al. [21]	2017	13	537	76.8% (71.8–81.4)	77.9% (71.9–83.2)	NR	NR	NR
	Juneau et al. [22]	2018	7	329	81% (73–86)	85% (78–91)	NR	NR	NR

CIED infections	Mahmood et al. [23]	2017	14	492	85% (80–89)	90% (84–94)	NR	NR	NR
	Juneau et al. [24]	2017	11	331	87% (82–91)	94% (88–98)	NR	NR	NR

Vascular graft infection	Reinders Folmer et al. [25]	2018	5	144	95% (87–99)	80% (69–89)	NR	NR	38 (8.5–170)
	Rojoa et al. [26]	2018	8	NR	97% (89–99)	89% (70–96)	NR	NR	NR

Cardiac sarcoidosis	Youssef et al.^*∗*^ [27]	2012	7	164	89% (79–96)	78% (68–86)	4.1 (1.7–10)	0.19 (0.1–0.4)	25.6 (7.3–89.5)
	Tang et al.^*∗*^ [28]	2016	16	559	75% (69–80)	81% (76–85)	NR	NR	16.9 (7.6–37.5)
	Kim et al.^*∗*^ [29]	2019	17	891	84% (71–91)	83% (74–89)	4.9 (3.3–7.3)	0.2 (0.11–0.35)	27 (14–55)

Osteomyelitis	Wang et al.^*∗*^ [30]	2011	7	319	92.3% (86.7–96.1)	92% (87–95.6)	9.8 (6–16)	0.11 (0.07–0.2)	98 (42.8–224)

Osteomyelitis related to diabetic foot	Treglia et al.^*∗*^ [31]	2013	4	178	74% (60–85)	91% (85–96)	5.6 (2–15.3)	0.37 (0.1–1.35)	16.9 (2–139.6)
	Lauri et al.^*∗*^ [32]	2017	6	254	89% (68–97)	92% (85–96)	11 (4.7–25)	0.11 (0.03–0.4)	95 (18–504)

Prosthetic joint infection	Jin et al.^*∗*^ [33]	2014	14	838	86% (82–90)	86% (83–89)	NR	NR	NR
	Verberne et al.^*∗*^ [34]	2016	10	666	86% (80–90)	93% (90–95)	NR	NR	NR
	Verberne et al.^*∗*^ [35]	2017	5	179	70% (56–81)	84% (76–90)	NR	NR	NR

Spondylodiscitis	Prodromou et al.^*∗*^ [36]	2014	12	224	97% (83–100)	88% (74–95)	8.2 (3.5–18.9)	0.03 (0–0.21)	NR
	Yin et al.^*∗*^ [37]	2018	6	191	96% (84–99)	90% (79–96)	9.8 (4.4–22)	0.05 (0.01–0.19)	124 (39–394)
	Kim et al.^*∗*^ [38]	2018	7	212	95% (87–98)	88% (73–95)	7.6 (3.4–17.2)	0.05 (0.02–0.14)	141 (44–444)

Rheumatic diseases	Descamps et al.^*∗*^ [39]	2018	90	2300	NR	NR	NR	NR	NR

Inflammatory bowel diseases	Treglia et al.^*∗*^ [40]	2013	7	219	85% (81–88)	87% (84–90)	6.2 (2.9–13.4)	0.19 (0.1–0.34)	44.3 (11.8–167)
	Zhang et al.^*∗*^ [41]	2014	3	162	84% (78–89)	86% (81–89)	5.3 (1.3–22)	0.2 (0.07–0.6)	25.9 (2.8–238)

LR+ = positive likelihood ratio; LR− = negative likelihood ratio; DOR = diagnostic odds ratio; 95% CI = 95% confidence interval; NR = not reported; CIED = cardiovascular implantable electronic device; GCA = giant cell arteritis; TA = Takayasu arteritis; TA^+^ = Takayasu arteritis using National Health Institute scale; ^*∗*^both PET and PET/CT are included.
